# Do Anti-SARS-CoV-2 Monoclonal Antibodies Have an Impact on Pregnancy Outcome? A Systematic Review and Meta-Analysis

**DOI:** 10.3390/vaccines11020344

**Published:** 2023-02-03

**Authors:** Ennio Conte, Raffaella Di Girolamo, Francesco D’Antonio, Antonio Raffone, Daniele Neola, Gabriele Saccone, Michela Dell’Aquila, Laura Sarno, Marco Miceli, Luigi Carbone, Giuseppe Maria Maruotti

**Affiliations:** 1 Department of Public Health, School of Medicine, University of Naples Federico II, 80131 Naples, Italy; 2 Center for Fetal Care and High-Risk Pregnancy, Department of Obstetrics and Gynecology, University of Chieti, 66100 Chieti, Italy; 3 Division of Gynaecology and Human Reproduction Physiopathology, Department of Medical and Surgical Sciences (DIMEC), IRCCS Azienda Ospedaliero-Universitaria di Bologna, Sant’Orsola Hospital, University of Bologna, Via Massarenti 13, 40138 Bologna, Italy; 4 Department of Neurosciences, Reproductive Sciences and Dentistry, School of Medicine, University of Naples Federico II, 80131 Naples, Italy; 5 CEINGE Biotecnologie Avanzate, 80145 Naples, Italy

**Keywords:** systematic review, COVID-19, SARS-CoV-2, monoclonal antibody (mAb), pregnancy outcome, preterm birth

## Abstract

Monoclonal antibodies (mAbs) have been used as a rescue strategy for pregnant women affected by COVID-19. To explore its impact on maternal-fetal health, we included all observational studies reporting maternal, fetal, delivery and neonatal outcomes in women who underwent mAbs infusion for COVID-19. Primary outcome was the percentage of preterm delivery. We used meta-analyses of proportions to combine data for maternal, fetal, delivery and neonatal outcome of women treated with mAbs for COVID-19 and reported pooled proportions and their 95% confidence intervals (CIs) for categorical variables or mean difference (MD) with their 95% confidence intervals for continuous variables. Preterm birth was observed in 22.8% of cases (95% CI 12.9–34.3). Fetal distress was reported in 4.2% (95% CI 1.6–8.2). Gestational hypertension and pre-eclampsia were observed in 3.0% (95% CI 0.8–6.8) and 3.4% (95% CI 0.8–7.5) of cases, respectively. Fetal growth restriction was observed in 3.2% of fetuses (95% CI 0.8–7.0). Secondary prophylaxis with mAbs is currently considered the best treatment option for people with mild to moderate COVID-19 disease. More attention should be paid to infants born from mothers who were treated with mAbs, for the risk of immunosuppression.

## 1. Introduction

The pandemic caused by severe acute respiratory syndrome coronavirus 2 (SARS-CoV-2) infection that was first identified in December 2019 remains a major public health issue [1,2]. Obstetrics and gynecology practice has undergone many changes during the last 2 years [3,4,5,6,7,8]. Furthermore, SARS-CoV-2 infection during pregnancy is associated with increased risk for adverse outcome compared to non-pregnant women. A multicenter study observed that 11.1% women were admitted to the ICU, 36 (9.3%) required mechanical ventilation and 3 (0.8%) died. In addition, 19.4% of the 31 women with first-trimester infection had miscarriage, 6 (2.3%) had stillbirth and 26.3% (70/266) had a preterm delivery [9]. Furthermore, SARS-CoV-2-infected women were found with hypertensive disorders in pregnancy (adjusted hazard ratio (aHR) 1.31, 95% CI 1.04–1.64), early pregnancy loss (aHR 1.37, 95% CI 1.00–1.88) and small-for-gestational-age children (aHR 1.28, 95% CI 1.05–1.54) [10]. Although the rate of cesarean section was often found to have increased, interestingly, with time this rate reduced and the induction of labor was more frequently performed [11]. In addition, a meta-analysis showed increased rates of placental histologic findings consistent with hypoperfusion and inflammation [12].

The need to get the vaccine during pregnancy has been long debated, and many reports have been released on the scarce acceptance rates demonstrated by diverse populations of pregnant women worldwide [13,14,15,16,17], although the rate of obstetric complications was shown not to be different to unvaccinated women [18]. National and international societies have produced guidelines and recommendations, initially considering high-risk pregnancies as the main indications for the vaccine and proposing to include pregnant women into future vaccine trials, and then assessing that pregnancy should not be considered a contraindication to the vaccine [19].

At the end of 2020, according to the United States Food and Drug Administration (FDA, Silver Spring, MD, USA), monoclonal antibodies (mAbs) had become a therapeutic option for non-hospitalized subjects with mild to moderate disease, to prevent the risk of developing a severe form of coronavirus disease 2019 (COVID-19) [20]. Additionally, recent data showed a partial benefit even in hospitalized patients [21]. Given that SARS-CoV-2 infection can lead to cytokine storm and the formation of auto-antibodies, responsible for tissue and vascular damage, with eventual pro-thrombotic consequences at the placental level, the use of monoclonal antibodies could also be seen as an option to reduce auto-immune response against maternal and or placental tissues [22]. In the last several decades, the use of mAbs during pregnancy to treat chronic autoimmune diseases has been increasing. In fact, in case of relapse during pregnancy, both maternal and fetal outcomes worsen. Trans-placental passage varies depending on the specific drug structure, half-life, dose and timing of the last dose, according to gestational age [23]. Usually, transfer during the first trimester is determined only by diffusion across the placenta, and is in low amounts. Later, during the second and third trimester, mAbs are actively transported, with a high rate of transfer after 36 weeks. IgG1 is the most efficiently transferred [24]. At birth, mAbs levels are usually higher in the newborn than in the mother, and there is an inverse correlation between the last maternal dose and cord blood concentration [25]. Data on pregnancy outcomes derive from retrospective observational cohort studies with small sample size, but there is no report of increased adverse outcomes. The largest study is the PIANO study, which evaluated the outcomes of pregnant women with inflammatory bowel disease: the rates of congenital malformation, miscarriage, preterm delivery, low birth weight and infant infection were not increased compared with the nonexposed group [26]. However, there are other reports of association between mAbs and preterm birth risk, since it was shown that women treated with anti-tumor necrosis factors (TNFs) had a higher risk of preterm birth compared to their healthy control [27]. In addition, concerns were raised about the effects of in utero mAbs therapy and neonatal of infant consequences: cytopenia has been observed, although transiently, while rates of infections are still questioned, and the use of live attenuated vaccines is to be avoided until 6–12 months of age or whenever drug levels become undetectable [28].

It is noteworthy that the efficacy of mAbs against COVID-19 consists in linking directly to the receptor-binding domain of spike protein (through which the virus may be able to recognize and bind the angiotensin-converting enzyme-2 (ACE2) receptor and enabling the virus to enter human cells), reducing both the mortality and the morbidity risk, as well as the length of hospitalization [29,30]. As a result, although no clinical trials on mAbs included pregnant women for ethical reasons, the National Institutes of Health (NIH) guidelines declared that the therapeutic approach for pregnant women with COVID-19 should be the same as for non-pregnant women, whereas FDA gave a warning signal for pregnant and lactating women because mAbs actively cross the placenta, causing an early immunodepression in infants [31]. Overall, data about the safety of anti–SARS-CoV-2 mAbs during pregnancy are still scarce [32].

The aim of this systematic review is to perform a cumulative analysis of pregnancy outcomes after mAbs infusion for COVID-19, to better understand their impact in pregnant women.

## 2. Materials and Methods

### 2.1. Search Strategy and Eligibility Criteria

This review was performed according to a protocol recommended for systematic review. The study was reported according to the Preferred Reporting Items for Systematic Reviews and Meta-analyses (PRISMA) guidelines [33] and Meta-analysis Of Observational Studies in Epidemiology (MOOSE) guidelines [34]. PRISMA and MOOSE checklists are reported in the Supplementary Materials (Supplementary Materials Tables S1 and S2). The review was registered in PROSPERO (CRD42022356986) before the start of the literature search. The literature search was conducted using Web of Science, Scopus, ClinicalTrials.gov, MEDLINE, Embase, Ovid and Cochrane Library as electronic databases. The studies were identified with the use of a combination of the following search terms: “coronavirus” OR “COVID-19 pandemic” OR “SARS-CoV-2” OR “COVID-19” AND “monoclonal” OR “monoclonal antibody” OR “mAb” OR “mAbs” AND “pregnancy” OR “pregnant” OR “pregnant women” OR “during pregnancy” OR “pregnancy outcome” OR “fetal outcome” OR “adverse outcome” from 1 December 2019 to 12 August 2022.

### 2.2. Study Selection

All review stages were conducted independently by two authors. In particular, two authors (E.C., R.D.G.) independently assessed the electronic search, eligibility of the studies, inclusion criteria, risk of bias and data extraction; R.D.G. performed data analysis. All disagreements were resolved by discussion with senior authors (L.C., G.M.M.). Review of articles also included the abstracts of all references retrieved from the search. Duplications were removed using Endnote online software as well as manually. Only studies written in English were considered for inclusion. Unpublished or non-peer-reviewed studies were not included. Given that for ethical reasons no randomized controlled studies were planned in pregnant women, we included in our systematic review all retrospective studies and case series that evaluated the population of women receiving monoclonal antibody therapy against SARS-CoV-2 during the period of COVID-19 pandemic and evaluated pregnancy, maternal and fetal-neonatal outcomes.

### 2.3. Data Extraction and Risk of Bias Assessment

A data extraction sheet based on the Cochrane data extraction template for non-RCTs was used (https://dplp.cochrane.org/data-extraction-forms) (last accessed on the 15 August 2022). The main data extracted for our systematic review were: first authors’ names and publication year, study design, study location, period considered in the analysis, sample size, inpatient or outpatient drug administration, type of intervention, vaccination for COVID-19, as well as various maternal, fetal and neonatal outcomes. Quality assessment of the included studies was performed using the Newcastle-Ottawa Scale (NOS) for case–control or cohort studies. According to the NOS, each study is judged on three broad perspectives: the selection of the study groups, the comparability of the groups and the ascertainment of outcome of interest. Assessment of the selection of a study includes the evaluation of the representativeness of the exposed cohort, selection of the non-exposed cohort, ascertainment of exposure and the demonstration that the outcome of interest was not present at the start of study. Assessment of the comparability of the study includes the evaluation of the comparability of cohorts based on the design or analysis. Finally, the ascertainment of the outcome of interest includes the evaluation of the type of assessment of the outcome of interest, its length and the adequacy of follow up. According to the NOS, a study can be awarded a maximum of one star for each numbered item within the Selection and Outcome categories. A maximum of two stars can be given for Comparability [35]. Case series were evaluated with a modified version of NOS [36], which is based on eight questions in the domains of selection, ascertainment, causality and reporting. Although a formal score could be assigned giving a binary response to each question, the numeric representation of methodological quality was not considered appropriate as recommended, and the overall final judgment was made based on questions 1, 2, 3, 7 and 8, which were deemed most critical in this specific clinical scenario (Supplementary Materials Table S3). We included case series/reports for which an adequate response to the abovementioned questions were found.

### 2.4. Data Synthesis

Outcomes were the evaluation of maternal, fetal, delivery and neonatal outcomes in pregnant women undergoing mAbs infusion. In particular, the primary outcome was the percentage of women who delivered preterm, defined as any birth before 37 completed weeks of gestation [37]. Other outcomes included the following: (1) adverse outcomes—adverse effect to infusion, fetal distress, gestational hypertension, pre-eclampsia, preterm premature rupture of membranes (pPROM), PROM, fetal growth restriction (FGR), cardiotocography (CTG) category III according to The International Federation of Gynecology and Obstetrics (FIGO) classification [38]; (2) delivery outcomes—preterm birth for COVID-19 maternal indication, full-term birth, vaginal delivery, operative delivery, urgent cesarean section, planned cesarean delivery, cesarean section, still pregnant; (3) neonatal outcomes—intensive care unit (ICU) admission, neonatal resuscitation, neonatal jaundice, neonatal death and 5 min Apgar < 7.

### 2.5. Statistical Analysis

We used meta-analyses of proportions to combine data. For categorical variables we reported pooled proportions and their 95% confidence intervals (CIs) for maternal, fetal and neonatal outcome of women treated with mAbs. For continuous variables, results were expressed as mean difference (MD) with their 95% confidence intervals. Data not reported as mean and standard deviation were transformed by appropriate statistical methods (e.g., by median and range) as described by Wan et al. [39]. All analyses were performed by adopting the random effect model of DerSimonian and Laird. Statistical heterogeneity among included studies was evaluated by the inconsistency index I2. In detail, heterogeneity was classified as null for I2 = 0%, minimal for I2 < 25%, low for I2 < 50%, moderate for I2 < 75% and high for I2 ≥ 75%. The analysis was performed using StatsDirect 3.0.171 (StatsDirect Ltd., Merseyside, UK) and Revman 5.3 (The Nordic Cochrane Centre, The Cochrane Collaboration, 2014) statistical software. A *p* value of <0.05 was considered significant.

## 3. Results

### 3.1. Study Selection and Characteristics

**Figure 1:**
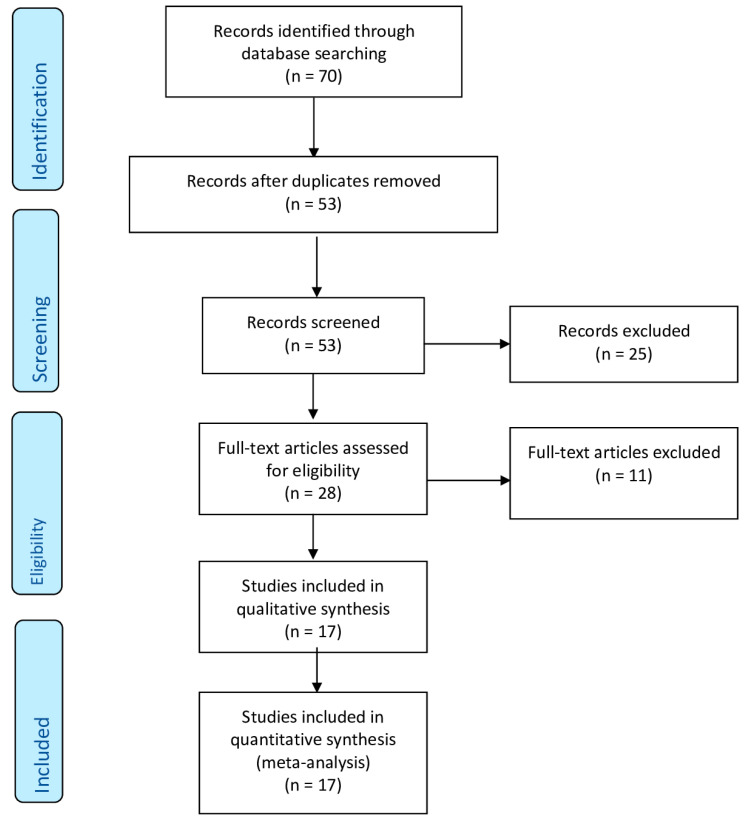
PRISMA flow diagram.

We identified 53 articles, 28 of which were assessed with respect to their eligibility for inclusion and 17 studies ultimately included in the systematic review (Table 1, Figure 1) [30,32,40,41,42,43,44,45,46,47,48,49,50,51,52,53,54]. Eight studies came from the United States of America (USA), four from Europe, three from Japan and the other two studies were from the United Arab Emirates (UAE). Monoclonal antibodies utilized in the present studies are presented in Table 2. Overall, these 17 studies included 190 pregnant women affected by mild to severe SARS-CoV-2 infection disease and treated with mAbs. The drug infusion was practiced at admission in the hospital for 81 patients and as outpatients for 105 other pregnant women. Hirshberg et al. [42] did not specify whether the treatment was administered as in- or outpatient for their four cases. Six studies included women also treated with anti-mRNA drugs for SARS-CoV-2 infection (Table 1). The results of the quality assessment of the included studies using NOS and NOS modified scale are presented in Supplementary Materials Table S4. The included studies showed an overall good score regarding the selection and comparability of the study groups, and for ascertainment of the outcome of interest. Excluded studies and reason for exclusion are reported in the Supplementary Materials (Supplementary Materials Table S5).

### 3.2. Synthesis of the Results

Maternal features are summarized in Table 3. Among adverse outcomes analyzed, 12.8% (95% CI 4.1–25.5) of women reported adverse effects to mAbs infusion. Fetal distress was observed in 4.2% (95% CI 1.6–8.2), while gestational hypertension and pre-eclampsia were observed in 3.0% (95% CI 0.8–6.8) and 3.4% (95% CI 0.8–7.5) of cases, respectively. In addition, pPROM and PROM were detected in 3.4% (95% CI 0.8–7.5) and 1.6% (95% CI 0.1–4.7) of patients, respectively, while fetal growth restriction was observed in 3.2% (95% CI 0.8–7.0) of fetuses. Furthermore, non-reassuring fetal status, expressed by CTG classification criteria (as class III) was reported in 7.4% (95% CI 3.4–12.6) of cases (Table 4) and stillbirth occurred in one fetus, despite already carrying Ebstein anomaly (Supplementary Materials Table S6). Moreover, regarding delivery outcome, preterm birth was observed in 22.8% (95% CI 12.9–34.3) of cases, although in 29.9% (95% CI 13.0–50.2) of these it was indicated by worsening maternal COVID-19 condition. Only one woman delivered shortly after mAb infusion: the patient experienced tachypnea, wheezing and shaking, followed by oxygen desaturation to 90%, fetal bradycardia followed by tachycardia to 210 beats per minute (Supplementary Materials Table S5). Overall, 48.4% (95% CI 40.0–56.9) of women delivered by vaginal delivery, while 4.6% (95% CI 1.6–9.0) of cases had operative delivery. Additionally, 12.6% (95% CI 7.6–18.4) of women had an urgent cesarean section, 15.6% (95% CI 7.8–25.7) of cases underwent a planned cesarean delivery, while 5.4% (95% CI 2.2–9.9) of women had a cesarean section without specified indication. Moreover, in 26.3% (95% CI 15.3–39.0) of cases women were reported as still pregnant (Table 5). Nonetheless, among neonatal outcomes, 15.9% (95% CI 8.0–26.0) of infants transiently went to the intensive care unit, while neonatal resuscitation was performed in 30.1% (95% CI 18.0–43.8) of cases. Furthermore, neonatal jaundice was observed in 26.7% (95% CI 0.5–72) of cases. We found that 2.2% (95% CI 0.6–4.7) of newborns died, and 5.9% (95% CI 0.4–17.1) of patients had a 5 min Apgar score < 7 (Table 6). One case of cytomegalovirus (CMV) re-activation was described: congenital CMV was confirmed by urine and blood determinations to the newborn. This patient received antiviral treatment from birth. Fundus examination, auditory evoked potentials, trans-fontanellar ultrasound and brain magnetic resonance imaging were normal. Furthermore, we calculated the composite adverse outcome, taking into consideration maternal, fetal and neonatal outcomes, observing that 36.9% (CI 21.0–54.4) of cases reported an adverse outcome.

## 4. Discussion

### 4.1. Main Findings

We found that 22.8% of women affected by SARS-CoV-2 infection and treated with mAbs had a preterm birth. However, a large proportion of these deliveries were carried out for worsening maternal conditions, and not for adverse effects related to mAbs treatment. Indeed, 12.8% of patients experienced side effects and 36.9% of cases reported an adverse maternal, fetal and/or neonatal outcome. Regarding neonatal outcome, we consider that an increased rate of complication could be the direct consequence of preterm delivery itself.

### 4.2. Strengths and Limitations

The main strengths of the present systematic review include a thorough literature search aimed at including all potentially relevant studies, adherence to PRISMA and MOOSE guidelines, the inclusion of all different mAbs used for SARS-CoV-2 infection during pregnancy, as well as the inclusion of a wide spectrum of outcomes. The retrospective design of the included studies, the small sample sizes and their heterogeneity are the main limitations. However, it should be once again noted that data are still scarce, as pregnant women have not been included in any of the trials performed on monoclonal antibodies and COVID-19 to date. Unfortunately, we could not stratify by virus variant or for vaccinated and unvaccinated women for SARS-CoV-2. Despite these limitations, the present systematic review represents the most comprehensive and up-to-date critical appraisal on the safety and pregnancy outcomes following monoclonal antibodies therapy for COVID-19 disease in pregnancy.

### 4.3. Implications and Future Perspectives

Overall, this study showed that monoclonal antibodies against SARS-CoV-2 might be considered a useful therapeutic option for pregnant women. These findings are also supported by NIH, the American College of Obstetricians and Gynecologists (ACOG) and the Society of Maternal-Fetal Medicine (SMFM) recommendations [55,56,57]. In particular, NIH guidelines allow the use of tocilizumab in pregnant women, acknowledging that these patients should be considered as non-pregnant women. Patients should be eligible for mAbs if they present within 10 days of symptom onset, with mild to moderate COVID-19 symptoms [55].

There is evidence of an association between preterm delivery and use of mAbs in pregnancy: treatment with anti-TNF showed a higher risk of preterm delivery, while infliximab was observed in association to a higher risk of small for gestational age (SGA) in women with inflammatory joint and skin diseases [27]. Moreover, Jorgensen et al. found increased preterm birth rate for tocilizumab (31.1%) compared to the general population (10–15%) [58]. Nonetheless, although 22.8% of women in our pooled analysis had preterm birth, it should be noted that almost 30% of these deliveries were indicated for worsening COVID-19 maternal conditions and only one case of delivery shortly after infusion was reported [39]. Similarly, Sekkarie et al. [59] observed a preterm birth rate of 29% in their cohort of pregnant women affected by COVID-19. Furthermore, in studies where a stratification of preterm birth in pregnancies affected by COVID-19 has been performed, it has been observed that the rate of preterm birth seems particularly increased only after 34 weeks [60,61].

Therefore, the possible impact of the disease itself and its clinical form on the choice to deliver the baby should not be underestimated, since it could be a possible confounding factor for the association between preterm birth and mAbs treatment. Close to term, obstetricians could be more prone to deliver the baby, considering the lower rate of complication for late preterm deliveries compared to earlier preterm. Moreover, Sekkarie et al. [59] described only antivirals and systemic steroids as the main therapies used for pregnant women, but not mAbs. Hence, these retrospective data could eventually show association but not directly a causative effect, for which differently designed trials should be planned. In fact, keeping in mind that our pooled analysis considered mostly case reports, case series and cohort studies, we cannot confirm a proper association between adverse outcomes and mAbs infusion, which could have come out from prospective studies. We only compared pregnant women receiving mAbs with others not receiving this treatment. Levey et al. [52] found no difference in obstetrical outcomes apart from increased incidence of cesarean section in the control group, while Magawa et al. [53] found different placental weights, although no differences were observed among the fetal-neonatal outcomes. In addition, recent evidence showed that mAbs used for treatment of other diseases such as multiple sclerosis do not carry increased risk for adverse pregnancy outcomes, both compared to unexposed pregnant women affected by MS and the general population [62].

Another important concern regarding mAbs infusion during pregnancy, given the existence of the transplacental passage between mother and fetus, is regarding eventual neonatal immunosuppression. Jiménez-Lozano et al. described the reactivation of CMV 8 days after admission in a patient who received tocilizumab. The presence of CMV was confirmed by urine and blood analyses of the newborn [44]. To overcome or at least control this issue, as an example, multiple sclerosis treatments with mAbs during pregnancy are suggested until 32–34 weeks of gestation, with the advice to monitor newborns for the risk of immunodepression and cytopenia [63]. However, such considerations could be inapplicable to COVID-19-affected pregnant women, given that it is an acute condition, where the risk of worsening should be counteracted by prompt management and treatment. It could be argued that if a woman is affected by COVID-19 at term, the first option would be an urgent delivery of the fetus (e.g., by cesarean section), allowing more aggressive treatments once the woman is no longer pregnant. However, there could be cases in which the prognosis of SARS-CoV-2 infection would be uncertain, and therefore the choice to immediately deliver the baby or not could be difficult, and a prophylactic therapy would be of help in reducing the rate of progression to a severe form of the infection. A recent study observed no difference by trimester of diagnosis in the frequency of COVID-19 disease progression in pregnancy [64]. Certainly, more attention should be paid to infants born from mothers who underwent mAbs therapy because of the risk of immunosuppression. In this regard, debate is also ongoing regarding the timing of live infant vaccines in these neonates [31].

Further studies and evidence should stratify the pregnant populations treated with mAbs according also to the virus variant, since the Omicron variant appears to be more resistant to mAbs action than the Delta variant, and according to the vaccination status. In addition, more research will be needed to evaluate the overall efficacy, as pregnant women should also be included in clinical trials of such monoclonals.

## 5. Conclusions

In our meta-analysis, 22.8% of pregnant women delivered preterm, but a substantial percentage of these early deliveries were due to worsening COVID-19 symptoms. Although it seems that the risk for adverse pregnancy outcomes is not increased, more data and longer follow-ups are needed. More attention should be paid for infants born from mothers who underwent mAbs therapy because of the risk of immune suppression.

## Figures and Tables

**Table 1:** General characteristics of included studies.

Authors, Year	Study Location	Study Design	Sample Size	Period Considered	Inpatients	Outpatients	Intervention	Anti-RNA	Outcomes
Abdullah et al., 2021 [40]	UAE	Case report	2	May 2020–June 2020	2	0	Single dose of tocilizumab 400 mg or 600 mg	yes	The impact of tocilizumab on COVID-19-related cytokine storm during pregnancy
Chang et al., 2022 [41]	USA	Retrospective study	30	December 2020–October 2021	10	20	Bamlanivimab (9); bamlanivimab/etesevimab (1); casirivimab/imdevimab (20)	no	The tolerability of infusion-related reactions; pregnancy outcomes
Hirshberg et al., 2021 [42]	USA	Retrospective study	4	November 2020–July 2021	ns	ns	Casirivimab/imdevimab	no	Additional COVID-19 care required; pregnancy outcome after infusion
Manciulli et al., 2022 [43]	Italy	Retrospective cohort study	8	1 March 2021–30 September 2021	6	2	Casirivimab/ imdevimab 2.4 g (patients not hospitalized for COVID-19) or casirivimab/imdevimab 8 g (patients hospitalized for COVID-19)	no	Gestational outcome (concluded pregnancy, complicated delivery, pregnancy in progress), presence of adverse reaction to monoclonal antibodies administration
Jiménez-Lozano et al., 2021 [44]	Spain	Retrospective study	12	1 March 2020–30 April 2020	12	0	Single or double dose of tocilizumab in lopinavir/ritonavir non-responder patients	yes	Adverse drug events related to tocilizumab administration in pregnant women and their offspring. Secondary outcomes were maternal and perinatal outcomes
Mayer et al., 2021 [30]	USA	Case report	2	NS	0	2	Casivirimab/imdevimab	no	NS
Richley et al., 2022 [45]	USA	Case series	15	1 April 2021–16 October 2021	12	3	Bamlanivimab/etesevimab; casirivimab/imdevimab	yes	Gestational outcome and presence of adverse reaction to monoclonal antibodies administration
Thilagar et al., 2021 [46]	USA	Retrospective cohort study	51	6 November 2020–30 October 2021	0	51	Casivirimab/imdevimab (44); bamlanivimab (4); bamlanivimab/etesevimab (3)	no	Additional COVID-19 care required, live births after infusion
Naqvi et al., 2020 [47]	USA	Case report	1	NS	1	0	Tocilizumab 400 mg followed by 5 days remdesivir 100 mg (200 mg first day)	yes	NS
Zöllkau et al., 2022 [48]	Germany	Case series	5	27 November 2021–1 December 2021	5	0	Casivirimab/imdevimab	no	Delivery
AlKindi et al., 2022 [32]	UAE	Case report	1	2021	1	0	Sotrovimab 500 mg	no	Delivery outcome, presence of adverse reaction to monoclonal antibodies administration
Ogawa et al., 2022 [49]	Japan	Case report	1	August 2021	1	0	Casivirimab/imdevimab	no	Delivery outcome
Waratani et al., 2020 [50]	Japan	Case report	1	February 2020	1	0	Tocilizumab 400 mg	yes	Delivery and neonatal outcome
Folkman et al., 2022 [51]	Sweden	Case series	7	May–November 2021	7	0	Casivirimab/imdevimab	no	Gestational, neonatal outcome, presence of adverse reaction to monoclonal antibodies administration
Levey et al., 2022 [52]	USA	Retrospective case–control study	36	August 2021–October 2021	9	27	Casivirimab/imdevimab	no	Gestational, neonatal outcome, presence of adverse reaction to monoclonal antibodies administration
Magawa et al., 2022 [53]	Japan	Retrospective case–control study	8	August 2021 and October 2021	8	0	Casivirimab/imdevimab 600 mg	no	Gestational, delivery outcome, presence of adverse reaction to monoclonal antibodies administration
Burwick et al., 2022 [54]	USA	Case series	6	April–August 2020	6	0	Eculizumab 1200 mg; additional doses of eculizumab were given per protocol if the patient remained hospitalized	yes	Delivery outcome

ADE, adverse drug event; COVID-19, coronavirus disease 2019; NS: not specified; UAE: United Arab Emirates; USA: United States of America.

**Table 2:** Monoclonal antibodies used in the included studies.

Authors, Years	Casirivimab/Imdevimab	Tocilizumab	Bamlanivimab	Bamlanivimab/Etesevimab	Sotrovimab	Eculizumab
Abdullah et al., 2021 [40]	0	2	0	0	0	0
Chang et al., 2022 [41]	20	0	9	1	0	0
Hirshberg et al., 2021 [42]	4	0	0	0	0	0
Manciulli et al., 2022 [43]	8	0	0	0	0	0
Jiménez-Lozano et al., 2021 [44]	0	12	0	0	0	0
Mayer et al., 2021 [30]	2	0	0	0	0	0
Richley et al., 2022 [45]	14	0	0	1	0	0
Thilagar et al., 2021 [46]	44	0	4	3	0	0
Naqvi et al., 2020 [47]	0	1	0	0	0	0
Zöllkau et al., 2022 [48]	5	0	0	0	0	0
AlKindi et al., 2022 [32]	0	0	0	0	1	0
Ogawa et al., 2022 [49]	1	0	0	0	0	0
Waratano et al., 2020 [50]	1	1	0	0	0	0
Folkman et al., 2022 [51]	7	0	0	0	0	0
Levey et al., 2022 [52]	36	0	0	0	0	0
Magawa et al., 2022 [53]	8	0	0	0	0	0
Burwick et al., 2022 [54]	0	0	0	0	0	6

**Table 3:** Maternal characteristics expressed as mean ± standard deviation (SD) or in terms of pooled proportions with their 95% confidence intervals (CIs).

Maternal Characteristics	Studies (*n*)	Cases (N or *n*/N)	Mean (SD) or Pooled Proportions (95% CI)	I^2^ (%)
Age	15	169	32.7 (3.2)	-
BMI	7	53	28.6 (1.80)	-
Gestational age at treatment	6	77	29.66 (3.33)	-
**Ethnicity**				
Asian	9	15/109	33.0 (9.0–64.3)	87%
White race	10	106/145	60.6 (32.1–85.5)	89.3%
Black race	10	21/145	10.2 (0.8–28.0)	82.8%
**Co-morbidities**				
Gestational diabetes	15	8/147	8.7 (2.8–17.2)	46.4%
Diabetes mellitus (I-II)	16	8/183	5.9 (2.1–11.4)	29.8%
Asthma	16	22/183	11.8 (5.5–20.0)	44.8%
Cardiovascular disease or hypertension	16	13/183	8.2 (3.2–15.0)	40.9%
BMI > 25	15	68/177	36.7 (19.9–55.3)	79.3%
Chronic lung disease	15	11/147	6.1 (1.3–14.3)	52.3%
Mental illness	16	32/183	9.8 (2.5–21.2)	72.6%
**Parity**				
Nulliparous	9	16/61	31.0 (14.4–50.6)	41.7%
Multiparous	10	46/63	67.9 (49.6–83.7)	37.1%
**Stratification according to trimester at treatment**				
1st Trimester	12	14/84	17.9 (10.8–26.5)	0%
2nd Trimester	12	26/84	34.2 (19.1–51.0)	47.9%
3rd Trimester	12	44/84	54.7 (39.2–69.9)	38.7%
**Severity of disease**				
Mild	11	12/45	25.1 (7.1–49.4)	65%
Moderate	11	16/45	39.5 (22.6–57.9)	35%
Severe	11	14/45	29.4 (13.1–49.0)	44.9%
**Vaccination for SARS-CoV-2**				
Fully or partially vaccinated	13	7/115	6.7 (2.0–14.5)	30.5%
Not vaccinated	13	108/115	93.0 (85.4–97.9)	30.5%

BMI, body mass index.

**Table 4:** Adverse outcomes expressed in terms of pooled proportions with their 95% confidence intervals (CIs).

Adverse Outcome	Studies (*n*)	Cases (*n*/N)	Pooled Proportions (95% CIs)	I^2^ (%)
Adverse effect to infusion	17	16/190	12.8 (4.1–25.5)	63.7%
Fetal distress	12	5/136	4.2 (1.6–8.2)	0%
Gestational hypertension	10	3/148	2.5 (0.6–5.6)	0%
Pre-eclampsia	10	2/120	3.0 (0.8–6.8)	0%
pPROM	10	4/150	3.4 (0.8–7.5)	14%
PROM	9	1/114	1.6 (0.1–4.7)	0%
Fetal growth restriction	9	3/121	3.2 (0.8–7.0)	0%
CTG category III *	13	10/168	7.4 (3.4–12.6)	14.7%
Composite adverse outcome	17	42/190	36.9% (21.0–54.4)	76.3%

CTG, cardiotocography; pPROM, preterm premature rupture of membranes; PROM, premature rupture of membranes. * According to FIGO classification.

**Table 5:** Delivery outcomes expressed as mean ± standard deviation (SD) or in terms of pooled proportions with their 95% confidence intervals (CIs).

Delivery Outcome	Studies (*n*)	Cases (*n*/N)	Pooled Proportions (95% CIs)	I^2^ (%)
Preterm birth	13	24/129	22.8 (12.9–34.3)	44.3%
Preterm birth for COVID-19 maternal indication	11	6/24	29.9 (13.0–50.2)	20%
Vaginal delivery	14	61/126	48.4 (40.0–56.9)	0%
Operative delivery	12	4/116	4.6 (1.6–9.0)	0%
Urgent cesarean section	14	15/136	12.6 (7.6–18.4)	0%
Planned cesarean delivery	12	13/98	15.6 (7.8–25.7)	25.9%
Cesarean section not specified	13	5/128	5.4 (2.2–9.9)	0%
Still pregnant	16	61/189	26.3 (15.3–39.0)	64%

COVID-19, coronavirus disease 2019.

**Table 6:** Neonatal outcome expressed as mean ± standard deviation (SD) or in terms of pooled proportions with their 95% confidence intervals (CIs).

Neonatal Outcome	Studies (n)	Cases (n/N)	Pooled Proportions (95% CI)	I^2^ (%)
Transient ICU	11	16/107	15.9 (8.0–26.0)	27.6%
Neonatal resuscitation	5	13/45	30.1 (18.0–43.8)	0%
Neonatal jaundice	3	2/14	26.7 (0.5–72)	46.7%
Neonatal death	15	2/187	2.2 (0.6–4.7)	0%
5 min Apgar < 7	7	2/68	5.9 (0.4–17.1)	39.8%

ICU, intensive care unit.

## Data Availability

No new data were created or analyzed in this study. Data sharing is not applicable to this article.

## Supplementary Materials

The following supporting information can be downloaded at: https://www.mdpi.com/article/10.3390/vaccines11020344/s1, Table S1: PRISMA checklist; Table S2: MOOSE checklist; Table S3: Quality assessment of the included studies according to Newcastle-Ottawa Scale (NOS) for cohort studies; Table S4: Tool for evaluating the methodological quality of case reports and case series; Table S5: Excluded studies and reasons for the exclusion; Table S6: Adverse side effects observed in the included studies.

## References

[1] Mohamadian M., Chiti H., Shoghli A., Biglari S., Parsamanesh N., Esmaeilzadeh A. (2021). COVID-19: Virology, biology and novel laboratory diagnosis. J. Gene Med..

[2] Carbone L., Conforti A., La Marca A., Cariati F., Vallone R., Raffone A., Buonfantino C., Palese M., Mascia M., Di Girolamo R. (2022). The negative impact of most relevant infections on fertility and assisted reproduction technology. Minerva Obstet. Gynecol..

[3] Carbone L., Esposito R., Raffone A., Verrazzo P., Carbone I.F., Saccone G. (2022). Proposal for radiologic diagnosis and follow-up of COVID-19 in pregnant women. J. Matern. Fetal Neonatal Med..

[4] Alviggi C., Esteves S.C., Orvieto R., Conforti A., La Marca A., Fischer R., Andersen C.Y., Bühler K., Sunkara S.K., Polyzos N.P. (2020). COVID-19 and assisted reproductive technology services: Repercussions for patients and proposal for individualized clinical management. Reprod. Biol. Endocrinol..

[5] Picarelli S., Conforti A., Buonfantino C., Vallone R., De Rosa P., Carbone L., Di Girolamo R., Strina I., Esteves S.C., Alviggi C. (2020). IVF during coronavirus pandemic: Who comes first? The POSEIDON viewpoint. Italian J. Gynaecol. Obstet..

[6] Gilroy L.C., Al-Kouatly H.B., Minkoff H.L., McLaren R.A. (2022). Changes in obstetrical practices during the 2020 COVID-19 pandemic. Am. J. Obstet. Gynecol. MFM.

[7] Carbone L., Raffone A., Sarno L., Travaglino A., Saccone G., Gabrielli O., Migliorini S., Sirico A., Genesio R., Castaldo G. (2022). Invasive prenatal diagnosis during COVID-19 pandemic. Arch. Gynecol. Obstet..

[8] Carbone L., Raffone A., Travaglino A., Sarno L., Conforti A., Gabrielli O., De Vivo V., De Rosa M., Migliorini S., Saccone G. (2022). Obstetric A&E unit admission and hospitalization for obstetrical management during COVID-19 pandemic in a third-level hospital of southern Italy. Arch. Gynecol. Obstet..

[9] WAPM (World Association of Perinatal Medicine) Working Group on COVID-19 (2021). Maternal and perinatal outcomes of pregnant women with SARS-CoV-2 infection. Ultrasound Obstet. Gynecol..

[10] Aabakke A.J.M., Petersen T.G., Wøjdemann K., Ibsen M.H., Jonsdottir F., Rønneberg E., Andersen C.S., Hammer A., Clausen T.D., Milbak J. (2023). Risk factors for and pregnancy outcomes after SARS-CoV-2 in pregnancy according to disease severity: A nationwide cohort study with validation of the SARS-CoV-2 diagnosis. Acta Obstet. Gynecol. Scand..

[11] Sirico A., Carbone L., Avino L., Buonfantino C., De Angelis M.C., Di Cresce M., Fabozzi A., Improda F.P., Legnante A., Riccardi C. (2022). Trends in caesarean section rate according to Robson group classification among pregnant women with SARS-CoV-2 infection: A single-center large cohort study in Italy. J. Clin. Med..

[12] Di Girolamo R., Khalil A., Alameddine S., D’Angelo E., Galliani C., Matarrelli B., Buca D., Liberati M., Rizzo G., D’Antonio F. (2021). Placental histopathology after SARS-CoV-2 infection in pregnancy: A systematic review and meta-analysis. Am. J. Obstet. Gynecol. MFM.

[13] Carbone L., Mappa I., Sirico A., Di Girolamo R., Saccone G., Di Mascio D., Donadono V., Cuomo L., Gabrielli O., Migliorini S. (2021). Pregnant women’s perspectives on severe acute respiratory syndrome coronavirus 2 vaccine. Am. J. Obstet. Gynecol. MFM.

[14] Mappa I., Luviso M., Distefano F.A., Carbone L., Maruotti G.M., Rizzo G. (2021). Women perception of SARS-CoV-2 vaccination during pregnancy and subsequent maternal anxiety: A prospective observational study. J. Matern. Fetal Neonatal Med..

[15] Carbone L., Di Girolamo R., Mappa I., Saccone G., Raffone A., Di Mascio D., De Vivo V., D’Antonio F., Guida M., Rizzo G. (2022). Worldwide beliefs among pregnant women on SARS-CoV-2 vaccine: A systematic review. Eur. J. Obstet. Gynecol. Reprod. Biol..

[16] Rawal S., Tackett R.L., Stone R.H., Young H.N. (2022). COVID-19 vaccination among pregnant people in the United States: A systematic review. Am. J. Obstet. Gynecol. MFM.

[17] Sutton D., D’Alton M., Zhang Y., Kahe K., Cepin A., Goffman D., Staniczenko A., Yates H., Burgansky A., Coletta J. (2021). COVID-19 vaccine acceptance among pregnant, breastfeeding, and nonpregnant reproductive-aged women. Am. J. Obstet. Gynecol. MFM.

[18] Carbone L., Trinchillo M.G., Di Girolamo R., Raffone A., Saccone G., Iorio G.G., Gabrielli O., Maruotti G.M. (2022). COVID-19 vaccine and pregnancy outcomes: A systematic review and meta-analysis. Int. J. Gynaecol. Obstet..

[19] Di Girolamo R., Khalil A., Rizzo G., Capannolo G., Buca D., Liberati M., Acharya G., Odibo A.O., D’Antonio F. (2022). Systematic review and critical evaluation of quality of clinical practice guidelines on the management of SARS-CoV-2 infection in pregnancy. Am. J. Obstet. Gynecol. MFM.

[20] Bassetti M., Giacobbe D.R., Bruzzi P., Barisione E., Centanni S., Castaldo N., Corcione S., De Rosa F.G., Di Marco F., Gori A. (2021). Clinical management of adult patients with COVID-19 outside intensive care units: Guidelines from the Italian society of anti-infective therapy (SITA) and the Italian society of pulmonology (SIP). Infect. Dis. Ther..

[21] RECOVERY Collaborative Group (2022). Casirivimab and imdevimab in patients admitted to hospital with COVID-19 (RECOVERY): A randomised, controlled, open-label, platform trial. Lancet.

[22] Sodagar A., Javed R., Tahir H., Razak S.I.A., Shakir M., Naeem M., Yusof A.H.A., Sagadevan S., Hazafa A., Uddin J. (2022). Pathological features and neuroinflammatory mechanisms of SARS-CoV-2 in the brain and potential therapeutic approaches. Biomolecules.

[23] Pham-Huy A., Top K.A., Constantinescu C., Seow C.H., El-Chaâr D. (2021). The use and impact of monoclonal antibody biologics during pregnancy. Can. Med Assoc. J..

[24] Malek A., Sager R., Kuhn P., Nicolaides K.H., Schneider H. (1996). Evolution of maternofetal transport of immunoglobulins during human pregnancy. Am. J. Reprod. Immunol..

[25] Julsgaard M., Christensen L.A., Gibson P.R., Gearry R.B., Fallingborg J., Hvas C.L., Bibby B.M., Uldbjerg N., Connell W.R., Rosella O. (2016). Concentrations of adalimumab and infliximab in mothers and newborns, and effects on infection. Gastroenterology.

[26] Mahadevan U., Long M.D., Kane S.V., Roy A., Dubinsky M.C., Sands B.E., Cohen R.D., Chambers C.D., Sandborn W.J., Crohn’s Colitis Foundation Clinical Research Alliance (2021). Pregnancy and neonatal outcomes after fetal exposure to biologics and thiopurines among women with inflammatory bowel disease. Gastroenterology.

[27] Bröms G., Kieler H., Ekbom A., Gissler M., Hellgren K., Lahesmaa-Korpinen A.M., Pedersen L., Schmitt-Egenolf M., Sørensen H.T., Granath F. (2020). Anti-TNF treatment during pregnancy and birth outcomes: A population-based study from Denmark, Finland, and Sweden. Pharmacoepidemiol. Drug Saf..

[28] Pham-Huy A., Sadarangani M., Huang V., Ostensen M., Castillo E., Troster S.M., Vaudry W., Nguyen G.C., Top K.A. (2019). From mother to baby: Antenatal exposure to monoclonal antibody biologics. Expert Rev. Clin. Immunol..

[29] Weinreich D.M., Sivapalasingam S., Norton T., Ali S., Gao H., Bhore R., Musser B.J., Soo Y., Rofail D., Im J. (2021). REGN-COV2, a Neutralizing antibody cocktail, in outpatients with Covid-19. N. Engl. J. Med..

[30] Mayer C., VanHise K., Caskey R., Naqvi M., Burwick R.M. (2021). Monoclonal antibodies casirivimab and imdevimab in pregnancy for Coronavirus Disease 2019 (COVID-19). Obstet. Gynecol..

[31] Burkhardt I., Whittaker E. (2022). Use of single-dose tocilizumab for treatment of severe COVID-19 in pregnancy: Implications for the timing of live infant vaccines. Arch. Dis. Child..

[32] AlKindi F., Chaaban A., Al Hakim M., Boobes Y. (2022). Sotrovimab use for COVID-19 infection in pregnant kidney transplant recipient. Transplantation.

[33] Liberati A., Altman D.G., Tetzlaff J., Mulrow C., Gotzsche P.C., Ioannidis J.P., Clarke M., Devereaux P.J., Kleijnen J., Moher D. (2009). The PRISMA statement for reporting systematic reviews and meta-analyses of studies that evaluate healthcare interventions: Explanation and elaboration. Ann. Intern. Med..

[34] Stroup D.F., Berlin J.A., Morton S.C., Olkin I., Williamson G.D., Rennie D., Moher D., Becker B.J., Sipe T.A., Thacker S.B. (2000). Meta-analysis of observational studies in epidemiology: A proposal for reporting. Meta-analysis of observational studies in epidemiology (MOOSE) group. JAMA.

[35] Lo C.K., Mertz D., Loeb M. (2014). Newcastle-Ottawa Scale: Comparing reviewers’ to authors’ assessments. BMC Med. Res. Methodol..

[36] Murad M.H., Sultan S., Haffar S., Bazerbachi F. (2018). Methodological quality and synthesis of case series and case reports. BMJ Evid. Based Med..

[37] Quinn J.A., Munoz F.M., Gonik B., Frau L., Cutland C., Mallett-Moore T., Kissou A., Wittke F., Das M., Nunes T. (2016). Preterm birth: Case definition & guidelines for data collection, analysis, and presentation of immunisation safety data. Vaccine.

[38] Safe Motherhood and Newborn Health Committee FIGO Consensus Guidelines on Intrapartum Fetal Monitoring. https://www.jsog.or.jp/international/pdf/CTG.pdf.

[39] Wan X., Wang W., Liu J., Tong T. (2014). Estimating the sample mean and standard deviation from the sample size, median, range and/or interquartile range. BMC Med. Res. Methodol..

[40] Abdullah S., Bashir N., Mahmood N. (2021). Use of intravenous tocilizumab in pregnancy for severe Coronavirus Disease 2019 pneumonia: Two case reports. J. Med. Case Rep..

[41] Chang M.H., Cowman K., Guo Y., Bao H., Bernstein P.S., Gendlina I., Nori P. (2022). A real-world assessment of tolerability and treatment outcomes of COVID-19 monoclonal antibodies administered in pregnancy. Am. J. Obstet. Gynecol..

[42] Hirshberg J.S., Cooke E., Oakes M.C., Odibo A.O., Raghuraman N., Kelly J.C. (2021). Monoclonal antibody treatment of symptomatic COVID-19 in pregnancy: Initial report. Am. J. Obstet. Gynecol..

[43] Manciulli T., Modi G., Campolmi I., Borchi B., Trotta M., Spinicci M., Lagi F., Bartoloni A., Zammarchi L. (2022). Treatment with anti-SARS-CoV-2 monoclonal antibodies in pregnant and postpartum women: First experiences in Florence, Italy. Infection.

[44] Jiménez-Lozano I., Caro-Teller J.M., Fernández-Hidalgo N., Miarons M., Frick M.A., Batllori Badia E., Serrano B., Parramon-Teixidó C.J., Camba-Longueira F., Moral-Pumarega M.T. (2021). Safety of tocilizumab in COVID-19 pregnant women and their newborn: A retrospective study. J. Clin. Pharm. Ther..

[45] Richley M., Rao R.R., Afshar Y., Mei J., Mok T., Vijayan T., Weinstein S., Pham C.U., Madamba J., Shin C.S. (2022). Neutralizing monoclonal antibodies for Coronavirus Disease 2019 (COVID-19) in pregnancy: A case series. Obstet. Gynecol..

[46] Thilagar B.P., Ghosh A.K., Nguyen J., Theiler R.N., Wick M.J., Hurt R.T., Razonable R.R., Ganesh R. (2022). Anti-spike monoclonal antibody therapy in pregnant women with mild-to-moderate Coronavirus Disease 2019 (COVID-19). Obstet. Gynecol..

[47] Naqvi M., Zakowski P., Glucksman L., Smithson S., Burwick R.M. (2020). Tocilizumab and remdesivir in a pregnant patient with Coronavirus Disease 2019 (COVID-19). Obstet. Gynecol..

[48] Zöllkau J., Reuken P.A., Schleußner E., Groten T. (2022). Monoclonal SARS-CoV-2 antibodies in pregnancy-a case series. Dtsch. Arztebl. Int..

[49] Ogawa E., Goto H., Ushimaru H., Matsuo A., Takeda S., Nishimura R., Hondo T., Takahashi T. (2022). Vaginal delivery after improvement in COVID-19 by monoclonal antibody treatment: A case report and literature review. J. Infect. Chemother..

[50] Waratani M., Ito F., Tanaka Y., Mabuchi A., Mori T., Kitawaki J. (2021). Severe Coronavirus Disease pneumonia in a pregnant woman at 25 weeks’ gestation: A case report. J. Obstet. Gynaecol. Res..

[51] Folkman R., Blennow O., Tovatt T., Pettersson K., Nowak P. (2022). Treatment of COVID-19 with monoclonal antibodies casirivimab and imdevimab in pregnancy. Infection.

[52] Levey N.H., Forrest A.D., Spielman D.W., Easley K.A., Dude C.M., Badell M.L. (2022). Outcomes of pregnant patients treated with REGEN-COV during the COVID-19 pandemic. Am. J. Obstet. Gynecol. MFM.

[53] Magawa S., Nii M., Maki S., Enomoto N., Takakura S., Maegawa Y., Osato K., Tanaka H., Kondo E., Ikeda T. (2022). Evaluation of the tolerability of monoclonal antibody therapy for pregnant patients with COVID-19. J. Obstet. Gynaecol. Res..

[54] Burwick R.M., Dellapiana G., Newman R.A., Smithson S.D., Naqvi M., Williams J., Wong M.S., Bautista M., Gaden A., Kazani S.D. (2022). Complement blockade with eculizumab for treatment of severe Coronavirus Disease 2019 in pregnancy: A case series. Am. J. Reprod. Immunol..

[55] National Institutes of Health (NIH) COVID-19 Treatment Guidelines. https://www.covid19treatmentguidelines.nih.gov/special-populations/pregnancy/?utm_source=site&utm_medium=home&utm_campaign=highlights.

[56] American College of Obstetricians and Gynecologists (ACOG). https://www.acog.org/clinical-information/physician-faqs/covid-19-faqs-for-ob-gyns-obstetrics.

[57] Society for Maternal-Fetal Medicine (SMFM). https://www.smfm.org/covidclinical.

[58] Jorgensen S.C.J., Lapinsky S.E. (2022). Tocilizumab for Coronavirus Disease 2019 in pregnancy and lactation: A narrative review. Clin. Microbiol. Infect..

[59] Sekkarie A., Woodruff R., Whitaker M., Kramer M.R., Zapata L.B., Ellington S.R., Meaney-Delman D.M., Pham H., Patel K., Taylor C.A. (2022). Characteristics and treatment of hospitalized pregnant women with COVID-19. Am. J. Obstet. Gynecol. MFM.

[60] Fallach N., Segal Y., Agassy J., Perez G., Peretz A., Chodick G., Gazit S., Patalon T., Ben Tov A., Goldshtein I. (2022). Pregnancy outcomes after SARS-CoV-2 infection by trimester: A large, population-based cohort study. PLoS ONE.

[61] Hughes B.L., Sandoval G.J., Metz T.D., Clifton R.G., Grobman W.A., Saade G.R., Manuck T.A., Longo M., Sowles A., Clark K. (2022). First- or second-trimester SARS-CoV-2 infection and subsequent pregnancy outcomes. Am. J. Obstet. Gynecol..

[62] Andersen J.B., Sellebjerg F., Magyari M. (2022). Pregnancy outcomes after early fetal exposure to injectable first-line treatments, dimethyl fumarate or natalizumab in Danish women with multiple sclerosis. Eur. J. Neurol..

[63] Krajnc N., Bsteh G., Berger T., Mares J., Hartung H.P. (2022). Monoclonal antibodies in the treatment of relapsing multiple sclerosis: An overview with emphasis on pregnancy, vaccination, and risk management. Neurotherapeutics.

[64] Schell R.C., Macias D.A., Garner W.H., White A.M., McIntire D.D., Pruszynski J., Adhikari E.H. (2022). Examining the impact of trimester of diagnosis on COVID-19 disease progression in pregnancy. Am. J. Obstet. Gynecol. MFM.

