# Supplementary material for: Do Anti-SARS-CoV-2 Monoclonal Antibodies Have an Impact on Pregnancy Outcome? A Systematic Review and Meta-Analysis
Source: Vaccines (Basel). 2023 Feb 3;11(2):344. doi: 10.3390/vaccines11020344 (PMC9962092; doi:10.3390/vaccines11020344)
Supplement: Supplementary file 1 [file vaccines-11-00344-s001.zip › vaccines-2170760-supplementary.pdf]

Table S1

Section/topic	#	Checklist item	Reported on page #
**TITLE**			
Title	1	Identify the report as a systematic review, meta-analysis, or both.	1
**ABSTRACT**			
Structured summary	2	Provide a structured summary including, as applicable: background; objectives; data sources; study eligibility criteria, participants, and interventions; study appraisal and synthesis methods; results; limitations; conclusions and implications of key findings; systematic review registration number.	1
**INTRODUCTION**			
Rationale	3	Describe the rationale for the review in the context of what is already known.	2
Objectives	4	Provide an explicit statement of questions being addressed with reference to participants, interventions, comparisons, outcomes, and study design (PICOS).	2
**METHODS**			
Protocol and registration	5	Indicate if a review protocol exists, if and where it can be accessed (e.g., Web address), and, if available, provide registration information including registration number.	2
Eligibility criteria	6	Specify study characteristics (e.g., PICOS, length of follow-up) and report characteristics (e.g., years considered, language, publication status) used as criteria for eligibility, giving rationale.	2
Information sources	7	Describe all information sources (e.g., databases with dates of coverage, contact with study authors to identify additional studies) in the search and date last searched.	2
Search	8	Present full electronic search strategy for at least one database, including any limits used, such that it could be repeated.	2
Study selection	9	State the process for selecting studies (i.e., screening, eligibility, included in systematic review, and, if applicable, included in the meta-analysis).	2
Data collection process	10	Describe method of data extraction from reports (e.g., piloted forms, independently, in duplicate) and any processes for obtaining and confirming data from investigators.	3
Data items	11	List and define all variables for which data were sought (e.g., PICOS, funding sources) and any assumptions and simplifications made.	3
Risk of bias in individual studies	12	Describe methods used for assessing risk of bias of individual studies (including specification of whether this was done at the study or outcome level), and how this information is to be used in any data synthesis.	3

Summary measures	13	State the principal summary measures (e.g., risk ratio, difference in means).	3
Synthesis of results	14	Describe the methods of handling data and combining results of studies, if done, including measures of consistency (e.g., I^2) for each meta-analysis.	3
Risk of bias across studies	15	Specify any assessment of risk of bias that may affect the cumulative evidence (e.g., publication bias, selective reporting within studies).	3
Additional analyses	16	Describe methods of additional analyses (e.g., sensitivity or subgroup analyses, meta-regression), if done, indicating which were pre-specified.	4
**RESULTS**			
Study selection	17	Give numbers of studies screened, assessed for eligibility, and included in the review, with reasons for exclusions at each stage, ideally with a flow diagram.	4
Study characteristics	18	For each study, present characteristics for which data were extracted (e.g., study size, PICOS, follow-up period) and provide the citations.	4
Risk of bias within studies	19	Present data on risk of bias of each study and, if available, any outcome level assessment (see item 12).	4
Results of individual studies	20	For all outcomes considered (benefits or harms), present, for each study: (a) simple summary data for each intervention group (b) effect estimates and confidence intervals, ideally with a forest plot.	4
Synthesis of results	21	Present results of each meta-analysis done, including confidence intervals and measures of consistency.	5
Risk of bias across studies	22	Present results of any assessment of risk of bias across studies (see Item 15).	5
Additional analysis	23	Give results of additional analyses, if done (e.g., sensitivity or subgroup analyses, meta-regression [see Item 16]).	5
**DISCUSSION**			
Summary of evidence	24	Summarize the main findings including the strength of evidence for each main outcome; consider their relevance to key groups (e.g., healthcare providers, users, and policy makers).	12
Limitations	25	Discuss limitations at study and outcome level (e.g., risk of bias), and at review-level (e.g., incomplete retrieval of identified research, reporting bias).	12-13
Conclusions	26	Provide a general interpretation of the results in the context of other evidence, and implications for future research.	14
**FUNDING**			
Funding	27	Describe sources of funding for the systematic review and other support (e.g., supply of data); role of funders for the systematic review.	NA

Table S2

Item No	Recommendation	Reported on Page No
Reporting of background should include		
1	Problem definition	2
2	Hypothesis statement	2
3	Description of study outcome(s)	2-3
4	Type of exposure or intervention used	2-3
5	Type of study designs used	2-3
6	Study population	2-3
Reporting of search strategy should include		
7	Qualifications of searchers (eg, librarians and investigators)	2-3
8	Search strategy, including time period included in the synthesis and key words	2-3
9	Effort to include all available studies, including contact with authors	2-3
10	Databases and registries searched	2
11	Search software used, name and version, including special features used (eg, explosion)	2-3
12	Use of hand searching (eg, reference lists of obtained articles)	2-3
13	List of citations located and those excluded, including justification	2-3
14	Method of addressing articles published in languages other than English	2-3
15	Method of handling abstracts and unpublished studies	2-3
16	Description of any contact with authors	2-3
Reporting of methods should include		
17	Description of relevance or appropriateness of studies assembled for assessing the hypothesis to be tested	2-3
18	Rationale for the selection and coding of data (eg, sound clinical principles or convenience)	2-3
19	Documentation of how data were classified and coded (eg, multiple raters, blinding and interrater reliability)	2-3

20	Assessment of confounding (eg, comparability of cases and controls in studies where appropriate)	2-3
21	Assessment of study quality, including blinding of quality assessors, stratification or regression on possible predictors of study results	2-3
22	Assessment of heterogeneity	2-3
23	Description of statistical methods (eg, complete description of fixed or random effects models, justification of whether the chosen models account for predictors of study results, dose-response models, or cumulative meta-analysis) in sufficient detail to be replicated	4
24	Provision of appropriate tables and graphics	6-12
Reporting of results should include		
25	Graphic summarizing individual study estimates and overall estimate	6-12
26	Table giving descriptive information for each study included	Table 1
27	Results of sensitivity testing (eg, subgroup analysis)	NA
28	Indication of statistical uncertainty of findings	NA
Reporting of discussion should include		
29	Quantitative assessment of bias (eg, publication bias)	6-12
30	Justification for exclusion (eg, exclusion of non-English language citations)	Suppl
31	Assessment of quality of included studies	Suppl
Reporting of conclusions should include		
32	Consideration of alternative explanations for observed results	12-13
33	Generalization of the conclusions (ie, appropriate for the data presented and within the domain of the literature review)	12-13
34	Guidelines for future research	12-13
35	Disclosure of funding source	14

Table S3

**Domains**	**Leading explanatory questions**
Selection	1. Does the patient(s) represent(s) the whole experience of the investigator (centre) or is the selection method unclear to the extent that other patients with similar presentation may not have been reported?
Ascertainment	2. Was the exposure adequately ascertained?<br>3. Was the outcome adequately ascertained?
Causality	4. Were other alternative causes that may explain the observation ruled out?<br>5. Was there a challenge/rechallenge phenomenon?<br>6. Was there a dose–response effect?<br>7. Was follow-up long enough for outcomes to occur?
Reporting	8. Is the case(s) described with sufficient details to allow other investigators to replicate the research or to allow practitioners make inferences related to their own practice?

Questions 4, 5 and 6 are mostly relevant to cases of adverse drug events.

Table S4

Authors, year	Selection	Comparability	Outcome
Chang et al., 2022	★	★	★
Hirshberg et al., 2021	★	★★	★★
Manciulli et al., 2022	★★★	★★	★★
Jiménez-Lozano et al., 2021	★	★	★
Thilagar et al., 2021	★★	★	★
Levey et al., 2022	★★★	★★	★★
Magawa et al., 2022	★	★	★

A study can be awarded a maximum of one star for each numbered item within the Selection and Outcome categories. A maximum of two stars can be given for Comparability

Table S5

Authors, year	Title	Reasons for the exclusion
San-Juan et al., 2020	Incidence and clinical profiles of COVID-19 pneumonia in pregnant women: A single-centre cohort study from Spain	No delivery and fetal outcome were reported
Hayashi et al., 2020	Gasless laparoendoscopic single-site surgery for management of unruptured tubal pregnancy in a woman with moderate COVID-19 pneumonia after administration of remdesivir and casirivimab-imdevimab: A case report	No delivery and fetal outcome were reported
Martínez-Sánchez et al., 2021	"Safety profile of treatments administered in COVID 19 infection in pregnant women"	Review article
Burkhardt et al., 2022	Use of single-dose tocilizumab for treatment of severe COVID-19 in pregnancy: implications for the timing of live infant vaccines	Letter to the editor
Jorgensen et al., 2021	Tocilizumab for coronavirus disease 2019 in pregnancy and lactation: a narrative review	Review article
Kreuzberger et al., 2021	SARS-CoV-2-neutralising monoclonal antibodies for treatment of COVID-19	Review article

RECOVERY Collaborative Group	Casirivimab and imdevimab in patients admitted to hospital with COVID-19 (RECOVERY): a randomised, controlled, open-label, platform trial	No delivery and fetal outcome were reported. They mentioned only that 25 women were pregnant
Gupta et al., 2022	"Use of Sotrovimab in a Pregnant Patient With COVID-19 Infection"	No delivery and fetal outcome were reported
Takayama et al., 2021	Severe COVID-19 Pneumonia in a 30-Year-Old Woman in the 36th Week of Pregnancy Treated with Postpartum Extracorporeal Membrane Oxygenation	The patient delivered before the treatments
Eid et al., 2022	Outpatient Use of Monoclonal Antibodies in Pregnant Individuals With Mild or Moderate Coronavirus Disease 2019 (COVID-19)	No delivery and fetal outcome were reported
Sekkarie et al., 2022	Characteristics and treatment of hospitalized pregnant women with Coronavirus Disease 2019, COVID-19	The study describes the vaccination status, treatment, and outcomes of hospitalized, symptomatic pregnant women with COVID-19

Table S6

Authors, Year	Sample size	Adverse event to infusion (n)	Details
Abdullah et al., 2021	2	0	
Chang et al., 2022	30	1	hypotension and dizziness, resolved with fluids
Hirshberg et al., 2021	4	0	
Manciulli et al., 2022	8	0	
Jiménez-Lozano et al., 2021	12	3	CMV reactivation in infant born, cytolytic hepatotoxicity
Mayer et al., 2021	2	0	
Richley et al., 2022	15	2	tachypnea, wheezing, and shaking, followed by oxygen desaturation to 90%, fetal bradycardia followed by tachycardia to 210 beats per minute; difficult breathing, mild hypertension, two consecutive fetal decelerations
Thilagar et al., 2021	51	0	
Naqvi et al., 2020	1	1	mild thrombocytosis
Zöllkau et al., 2022	5	0	
AlKindi et al., 2022	1	0	
Ogawa et al., 2022	1	0	

Waratano et al., 2020	1	0	
Folkman et al., 2022	7	0	
Levey et al., 2022	36	1	shortness of breath and new oxygen requirement
Magawa et al., 2022	8	8	fever
Burwick et al., 2022	6	0	
